# Synthesis, Characterization, Absorption Properties, and Electronic Structures of Paddlewheel-Type Dirhodium(II) Tetra-μ-(*n*-naphthoate) Complexes: An Experimental and Theoretical Study

**DOI:** 10.3390/molecules24030447

**Published:** 2019-01-27

**Authors:** Yusuke Kataoka, Raiki Fukumoto, Natsumi Yano, Daiki Atarashi, Hidekazu Tanaka, Tatsuya Kawamoto, Makoto Handa

**Affiliations:** 1 Department of Chemistry, Interdisciplinary Graduate School of Science and Engineering, Shimane University, 1060 Nishikawatsu, Matsue, Shimane 690-8504, Japan; s179220@matsu.shimane-u.ac.jp (R.F.); s179802@matsu.shimane-u.ac.jp (N.Y.); atarashi@riko.shimane-u.ac.jp (D.A.); hidekazu@riko.shimane-u.ac.jp (H.T.); 2 Department of Chemistry, Faculty of Science, Kanagawa University, 2946 Tsuchiya, Hiratsuka, Kanagawa 259-1293, Japan; kaw@kanagawa-u.ac.jp

**Keywords:** dirhodium complex, absorption properties, crystal structure, redox properties

## Abstract

The reactions of [Rh_2_(O_2_CCH_3_)_4_(OH_2_)_2_] with *n*-naphthalenecarboxylic acids (*n* = 1: 1-HNC, *n* = 2: 2-HNC) afford the dirhodium tetra-μ-(*n*-naphthoate) complexes [Rh_2_(1-NC)_4_] (**1**) and [Rh_2_(2-NC)_4_] (**2**), respectively. Single crystal X-ray diffraction analyses of [1(OCMe_2_)_2_] and [2(OCMe_2_)_2_], which were obtained by recrystallization from acetone (OCMe_2_) solutions of **1** and **2**, reveal that the dirhodium cores are coordinated by four equatorially bridging naphthoate ligands and two axial OCMe_2_ ligands. Density functional theory (DFT) calculation confirmed that (i) the single Rh–Rh bond is formed between the two Rh ions and (ii) the electronic structures between two Rh ions in **[1(OCMe_2_)_2_]** and **[2(OCMe_2_)_2_]** are best described as π^4^δ^2^σ^2^δ*^2^π*^4^ and δ^2^π^4^σ^2^δ*^2^π*^4^, respectively. Time-dependent DFT (TDDFT) calculations clarify the absorption band characters of **[1(OCMe_2_)_2_]** and **[2(OCMe_2_)_2_]**; the former shows the bands due to d–d and metal–to–metal-ligand charge transfer (MMLCT) excitations in the visible light region, whereas the latter shows the bands due to only d–d excitations in the same region. The electrochemical properties and thermal stabilities of **[1(OCMe_2_)_2_]** and **[2(OCMe_2_)_2_]** were also investigated in this study.

## 1. Introduction

The chemistry of paddlewheel-type dirhodium complexes containing a Rh–Rh single [1] bond has attracted considerable attention because of their unique functional properties, which have been successfully employed in chemical sensors [2,3], antitumor agents [4], and catalysts for various types of organic reactions [5,6], and photochemical hydrogen evolution from aqueous solution [7]. The intriguing properties of dirhodium complexes are derived from specific molecular orbital (MO) interactions (e.g., σ^2^π^4^δ^2^π*^4^δ*^2^) between the two rhodium ions [8,9]. Further, on account of their chemical and water stabilities, these types of complexes have been recently utilized as robust building blocks for supramolecular complexes [10,11,12], coordination cages [13,14], coordination polymers [15,16,17,18], and porous metal organic frameworks (MOFs) [19,20,21,22]. The conventional synthetic strategy for paddlewheel-type dirhodium complexes involves a ligand-exchange reaction between dirhodium tetraacetate and organic carboxylic acids, at high temperature under inert gas. In this way, a large number of paddlewheel-type dirhodium complexes have been synthesized and characterized. However, the vast majority of these dirhodium complexes have aliphatic carboxylate or benzoate derivatives. Remarkably, acene carboxylate–coordinated dirhodium complexes have not yet been developed, whereas some paddlewheel-type dinuclear complexes (such as Cu_2_ complexes) with acene carboxylate, which, in crystalline solids, form supramolecular π–π stacking interactions between neighboring molecules [23,24], are already reported. Therefore, the development of paddlewheel-type dirhodium complexes with acene carboxylate, and detailed investigation of their electronic structures, absorption, and redox properties, via combination of experimental and theoretical techniques, provide meaning in the viewpoints of further expansion and the establishment of knowledge for this type of complex. Of course, the enlarged π-system is considered to be advantageous to synthesize chemically-stable supramolecular complexes and oxidation catalysts with dirhodium cores.

**Figure 1:**
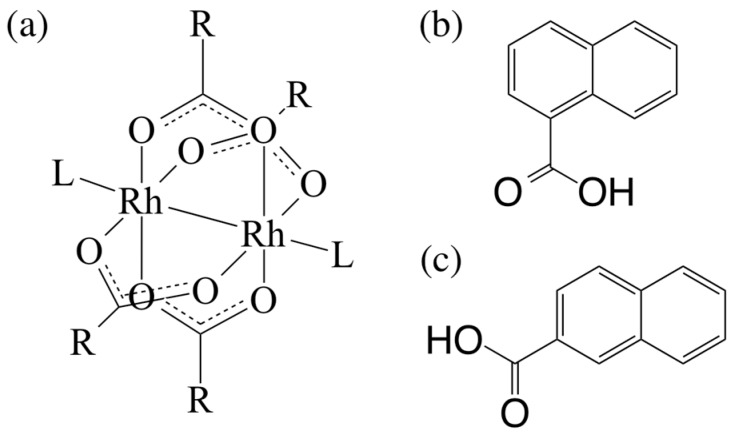
Molecular structures of: (**a**) [Rh_2_(O_2_CR)_4_L_2_], (**b**) 1-HNC, and (**c**) 2-HNC. Here, R and L moieties in [Rh_2_(O_2_CR)_4_L_2_] are alkyl (or aromatic) functional groups and axial-coordinated molecules, respectively.

In this study, we describe the synthesis of new paddlewheel-type dirhodium tetra-μ-(*n*- naphthoate) complexes [Rh_2_(*n*-NC)_4_] (*n* = 1 and 2; *n*-NC = *n*-naphthate), via the solvothermal reactions between [Rh_2_(O_2_CCH_3_)_4_(H_2_O)_2_] and corresponding *n*-naphthalenecarboxylic acids (*n*-HNC; see Figure 1). The obtained dirhodium complexes, which are recrystallized from acetone (OCMe_2_) (i.e., [Rh_2_(1-NC)_4_(OCMe_2_)_2_] (**[1(OCMe_2_)_2_]**) and [Rh_2_(2-NC)_4_(OCMe_2_)_2_] (**[2(OCMe_2_)_2_]**)) were fully characterized via single crystal X-ray diffraction analyses, ^1^H NMR spectroscopy, electrospray ionization mass spectrometry (ESI-MS), infrared spectroscopy, and elemental analyses. The absorption spectral features of these dirhodium complexes were investigated in detail by the help of time-dependent density functional theory (TDDFT) calculations. In addition, the electrochemical properties and thermal stabilities of **[1(OCMe_2_)_2_]** and **[2(OCMe_2_)_2_]** were also investigated.

## 2. Results and Discussions

### 2.1. Synthesis and Characterization of **[1(OCMe_2_)_2_]** and **[2(OCMe_2_)_2_]**

**[1(OCMe_2_)_2_]** and **[2(OCMe_2_)_2_]** were obtained as green powders in relatively high yields (90.6 and 89.1%, respectively), using a conventional ligand-exchange reaction between [Rh_2_(O_2_CCH_3_)_4_(H_2_O)_2_] and an excess amount of 1-HNC for **1** or 2-HNC for **2**, in degassed EtOH, under solvothermal conditions (at 413 K) for 7 h, followed by recrystallization from an acetone solution. The amount of minor by-products and heteroleptic complexes (i.e., [Rh_2_(*n*-NC)_m_(O_2_CCH_3_)_4-m_] (m = 1~3)) increased as the reaction time ws shorter, and a rhodium colloid was co-generated when the reaction time was increased. The desired homoleptic complexes were not obtained when the reaction temperature was lower than 373 K. When the reaction temperature was higher than 443 K; however, the rhodium colloid was co-generated. Decreasing the amount of the *n*-HNC ligands affords the corresponding heteroleptic dirhodium complexes. On the basis of these results, we concluded that the procedure described in this study was suitable to synthesize **[1(OCMe_2_)_2_]** and **[2(OCMe_2_)_2_]**. The obtained powders of **[1(OCMe_2_)_2_]** and **[2(OCMe_2_)_2_]** were stable in air and can be dissolved in chloroform (CHCl_3_), pyridine (Py), and dimethylsulfoxide (DMSO). They can also be dissolved slightly in acetone but not in water. Hence, the spectroscopic and electrochemical analyses of **[1(OCMe_2_)_2_]** and **[2(OCMe_2_)_2_]** were performed using an acetone:chloroform (1:1, *v*/*v*) mixture because of the improved solubility of the complexes.

ESI-MS of **[1(OCMe_2_)_2_]** and **[2(OCMe_2_)_2_]** in positive ion mode showed intense signals at 912.9783 and 912.9775 *m*/*z*, respectively; these values were consistent with the simulated [M + Na]^+^ values (912.9786 *m*/*z*) of **1** and **2**, which were axial acetone-dissociated complexes of **[1(OCMe_2_)_2_]** and **[2(OCMe_2_)_2_]**. As shown in Figures S1 and S2, the observed isotopic signal patterns of **1** and **2** can be well fitted to the simulated patterns of [M + Na]^+^ for **1** and **2**, respectively. The ^1^H NMR spectra of **[1(OCMe_2_)_2_]** and **[2(OCMe_2_)_2_]** in acetone-*d*_6_ showed 28 proton signals that were consistent with the total number of protons in the four *n*-NC ligands; superfluous proton signals, which were due to the presence of heteroleptic dirhodium complexes or unreacted *n*-HNC ligands, were not observed. The purities of **[1(OCMe_2_)_2_]** and **[2(OCMe_2_)_2_]** were also verified by elemental analysis; the observed C and H values were in good agreement with those calculated for **[1(OCMe_2_)_2_]** and **[2(OCMe_2_)_2_]**, with no superfluous solvents. These results indicate that heteroleptic complexes and unwanted by-products were not mixed into the obtained samples of **[1(OCMe_2_)_2_]** and **[2(OCMe_2_)_2_]**. In the infrared spectra, symmetric (ν_sym_(CO_2_^–^)) and asymmetric (ν_asym_(CO_2_^–^)) vibrational modes of the bridging carboxylate ligands of **[1(OCMe_2_)_2_]** were observed at 1561 and 1680 cm^−1^, respectively; these values were essentially identical with those of **[2(OCMe_2_)_2_]** (1563 and 1680 cm^−1^, respectively). The separation values, Δν_sym_(CO_2_^–^), between ν_sym_(CO_2_^–^) and ν_asym_(CO_2_^–^) of **[1(OCMe_2_)_2_]** and **[2(OCMe_2_)_2_]**, were 119 and 117 cm^−1^, respectively, which were similar to those of the typical dirhodium(II,II) complexes [1]. Raman spectra of **[1(OCMe_2_)_2_**] and **[2(OCMe_2_)_2_]** showed the intense Rh–Rh vibrations at 315 and 333 cm^−1^, respectively, indicating that **[1(OCMe_2_)_2_]** and **[2(OCMe_2_)_2_]** had stable Rh–Rh bonds in their structures. To investigate the thermal stabilities of **[1(OCMe_2_)_2_]** and **[2(OCMe_2_)_2_]**, TG-DTA analyses were performed. Figures S3 and S4 show the TG-DTA profiles of **[1(OCMe_2_)_2_]** and **[2(OCMe_2_)_2_]** under air, respectively. The weight losses of **[1(OCMe_2_)_2_]** and **[2(OCMe_2_)_2_]**, which were caused by the desorption of axial coordinated acetone molecules (calcd. weight = 11.5%), occurred at 383~413 K (10.1%) and 343~435 K (10.4%), respectively. The difference in the desorption temperature indicates that the axial coordinated acetone molecules in **[1(OCMe_2_)_2_]** were more strongly coordinated and/or tightly stacked in packing space than those in **[2(OCMe_2_)_2_]**. The decomposition of each [Rh_2_(n-NC)_4_] occurred above 573 K, which was almost identical to that of [Rh_2_(O_2_CPh)_4_(OCMe_2_)_2_] (563 K).

### 2.2. X-ray Diffraction Analyses of **[1(OCMe_2_)_2_]** and **[2(OCMe_2_)_2_]**

**Figure 2:**
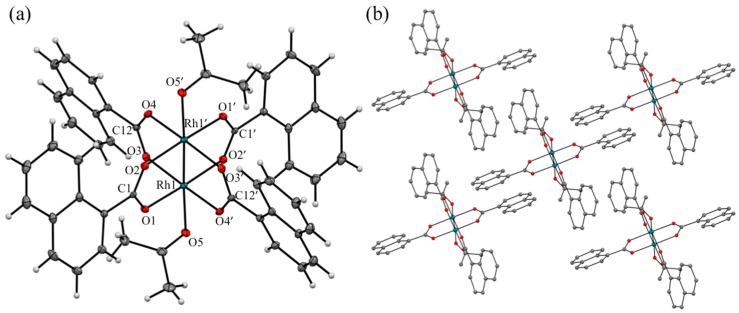
(**a**) ORTEP diagram (thermal ellipsoid: 30%); and (**b**) expanded surrounding structure of **[1(OCMe_2_)_2_]** crystal. In (**b**), hydrogen atoms are omitted for clarity.

**Figure 3:**
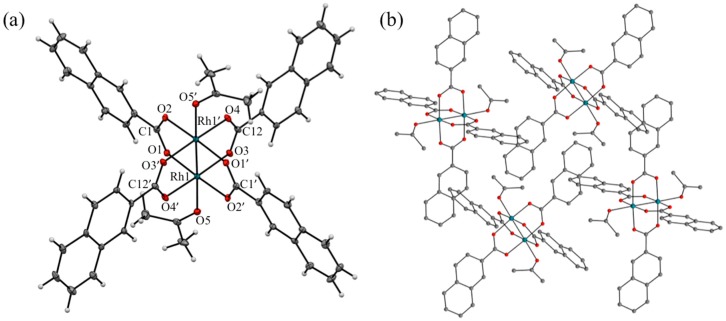
(**a**) ORTEP diagram (thermal ellipsoid: 30%); and (**b**) expanded surrounding structure of **[2(OCMe_2_)_2_]** crystal. In (**b**), hydrogen atoms are omitted for clarity.

Single crystal X-ray diffraction analyses were performed using small single crystals of **[1(OCMe_2_)_2_]** and **[2(OCMe_2_)_2_]**, which were recrystallized from acetone. The crystallographic data are summarized in Table 1, selected (averaged) structural parameters are listed in Table 2, and the ORTEP (30% ellipsoid) and packing structures of **[1(OCMe_2_)_2_]** and **[2(OCMe_2_)_2_]** are depicted in Figure 2 and Figure 3, respectively. Complexes **[1(OCMe_2_)_2_]** and **[2(OCMe_2_)_2_]** were crystallized in triclinic (space group: *P*-1) and monoclinic (space group: *P* 2_1_/*c*) crystal systems, respectively, and both complexes were closely assembled without void spaces in each crystal; that is the reason why, their crystals did not possess guest solvents (e.g., acetone or water).

In the asymmetric units of **[1(OCMe_2_)_2_]** and **[2(OCMe_2_)_2_]**, both structures contain one half of a molecule, comprising one rhodium ion, two naphthoate ligands, and one acetone ligand. Thus, a crystallographic inversion center is located at the middle of the Rh–Rh bond. Both complexes formed typical paddlewheel-type dinuclear structures, in which the dirhodium core was coordinated by four equatorial bridging naphthoate ligands and two axial acetone ligands. The primary coordination spheres of the rhodium ions in **[1(OCMe_2_)_2_]** and **[2(OCMe_2_)_2_]** were distorted octahedral. Bond length differences of the primary coordination spheres between **[1(OCMe_2_)_2_]** and **[2(OCMe_2_)_2_]** were negligibly small, and their bond lengths were in the ranges for typical paddlewheel-type dirhodium complexes, coordinated with *O*-donor solvent ligands (such as H_2_O and MeOH) at the axial positions. The Rh–Rh bond lengths of **[1(OCMe_2_)_2_]** and **[2(OCMe_2_)_2_]** were determined as 2.374 and 2.395 Å, respectively, which were rather shorter than that of [Rh_2_(O_2_CCF_3_)_4_(OCMe_2_)_2_] (2.406 Å) [25]. The averaged Rh–O_equatorial_ bond lengths of **[1(OCMe_2_)_2_]** (2.035 Å) and **[2(OCMe_2_)_2_]** (2.040 Å) were almost identical to that of [Rh_2_(O_2_CCF_3_)_4_(OCMe_2_)_2_] (2.036 Å), whereas the Rh–O_axial_ bond lengths of **[1(OCMe_2_)_2_]** (2.296 Å) and **[2(OCMe_2_)_2_]** (2.303 Å) were slightly longer than that of [Rh_2_(O_2_CCF_3_)_4_(OCMe_2_)_2_] (2.252 Å). These results suggest that the electron-withdrawing properties of the two naphthoate ligands were relatively weaker than that of the trifluoroacetate ligand. Regarding the dihedral angles defined by the naphthalene rings and carboxylate groups (θ_da_), an obvious difference was observed between **[1(OCMe_2_)_2_]** and **[2(OCMe_2_)_2_]**. The naphthalene rings in **[1(OCMe_2_)_2_]** were rotated with respect to the carboxylate moieties (θ_da_: 14.55° and 26.32°), whereas those in **[2(OCMe_2_)_2_]** were almost coplanar (6.83° and 9.30°). It is deduced that the dihedral angle differences between **[1(OCMe_2_)_2_]** and **[2(OCMe_2_)_2_]** originated from the different structural repulsions between naphthalene rings and the acetone ligand, and different intermolecular π–π stacking features of naphthalene rings in the crystal between **[1(OCMe_2_)_2_]** and **[2(OCMe_2_)_2_]**; rather than from the different effects of the electronic structures of the dinuclear cores of **[1(OCMe_2_)_2_]** and **[2(OCMe_2_)_2_]**.

In the packing structure of **[1(OCMe_2_)_2_]**, all four naphthalene rings in **[1(OCMe_2_)_2_]** individually formed intermolecular π–π stacking interactions with the naphthalene rings of four neighboring **[1(OCMe_2_)_2_]** moieties, as shown in Figure 2b. As a result, **[1(OCMe_2_)_2_]** formed two-dimensional supramolecular layers that self-assembly stacked in an AA fashion. The intermolecular π–π stacking distances were estimated to be 3.301 and 3.549 Å. In contrast, several intermolecular hydrogen bonding interactions were formed in the packing structure of **[2(OCMe_2_)_2_]**, though no intermolecular π–π interactions were formed (see Figure 3b).

**Figure 4:**
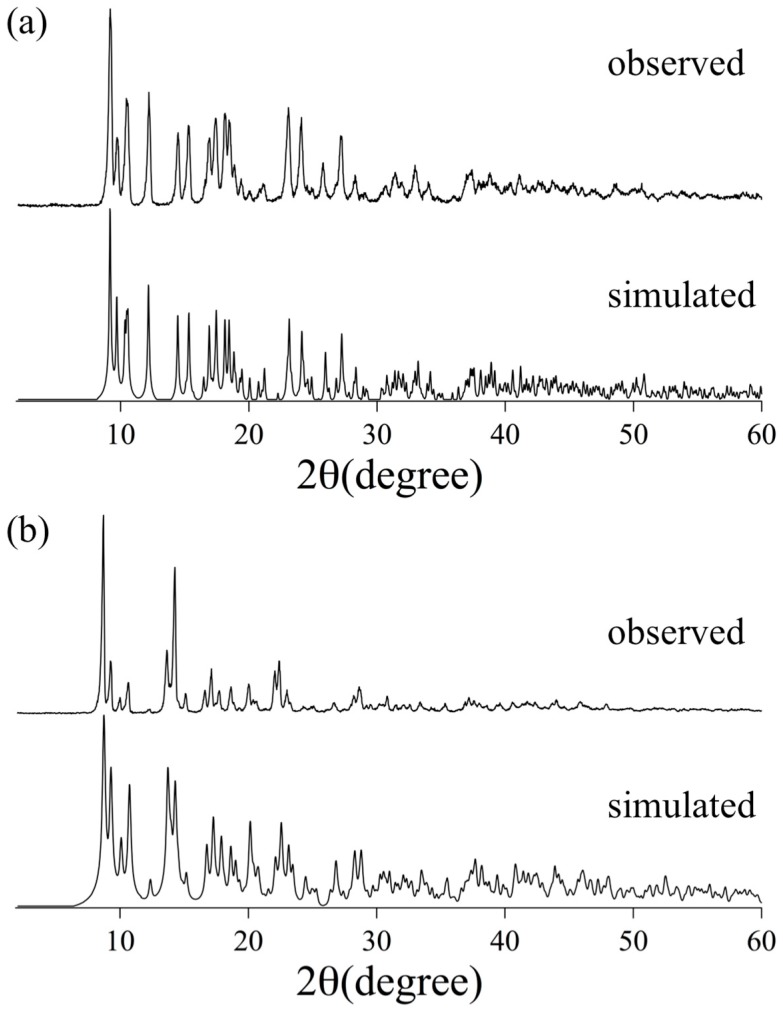
Observed and simulated X-ray powder diffraction (XRPD) patterns: (**a**) **[1(OCMe_2_)_2_]**; and (**b**) **[2(OCMe_2_)_2_]**.

In order to confirm the phase purities of **[1(OCMe_2_)_2_]** and **[2(OCMe_2_)_2_]**, X-ray powder diffraction analyses (XRPD) were performed. As depicted in Figure 4, the diffraction patterns of **[1(OCMe_2_)_2_]** and **[2(OCMe_2_)_2_]** were in good agreement with the simulated XRPD patterns of their crystal structures. These results clearly indicated that the crystalline powders of **[1(OCMe_2_)_2_]** and **[2(OCMe_2_)_2_]** were of single phase, and that the axial-coordinated acetone ligands were not dissociated by drying them by evaporation in their synthesis process.

### 2.3. Optimized Geometries and Electronic Structures of **[1(OCMe_2_)_2_]** and **[2(OCMe_2_)_2_]**

To clarify the molecular geometries and electronic structures of **[1(OCMe_2_)_2_]** and **[2(OCMe_2_)_2_]** in acetone media, DFT (functional method: B3LYP) calculations were performed using the PCM solvation method [26]. Figure S5 depicts the optimized geometries of **[1(OCMe_2_)_2_]** and **[2(OCMe_2_)_2_]**. Similarly to the experimentally observed structures, the optimized geometries of **[1(OCMe_2_)_2_]** and **[2(OCMe_2_)_2_]** had typical paddlewheel-type structures, in which the *n*-NC ligands and acetone molecules were coordinated in equatorial and axial coordination sites of the dirhodium units, respectively. Regarding the naphthyl rings, the θ_da_ values of the optimized geometry of **[1(OCMe_2_)_2_]** (averaged θ_da_: 36.94°) indicated a greater rotation angle than that observed in the experimentally-obtained **[1(OCMe_2_)_2_]** structure (θ_da_: 14.55° and 26.32°), whereas that of **[2(OCMe_2_)_2_]** (averaged θ_da_: 4.33°) was almost identical to that of the experimental **[2(OCMe_2_)_2_]** structure (6.83° and 9.30°). It is speculated that this difference was caused by weaker intermolecular π–π stacking interactions between neighboring naphthalene rings in the **[1(OCMe_2_)_2_]** crystal structure.

The primary coordination spheres of the optimized geometries of **[1(OCMe_2_)_2_]** and **[2(OCMe_2_)_2_]** reproduced their experimentally-observed structures rather well, and their structural parameters were almost identical. Specifically, the Rh–Rh bond lengths of the optimized geometries of **[1(OCMe_2_)_2_]** and **[2(OCMe_2_)_2_]** were both 2.415 Å, which was only 0.041 and 0.020 Å longer than the corresponding bonds in the experimentally-obtained **[1(OCMe_2_)_2_]** and **[2(OCMe_2_)_2_]** structures. The Rh–O_axial_ (in OCMe_2_) bond lengths of the optimized geometries of **[1(OCMe_2_)_2_]** and **[2(OCMe_2_)_2_]** were 2.358 and 2.359 Å, respectively; we deduce that the longer bond lengths (by ca. 0.06 Å) of the experimental **[1(OCMe_2_)_2_]** and **[2(OCMe_2_)_2_]** structures, than those of the theoretical ones, originated from crystal packing stress. No remarkable differences were observed in the Rh–O_equatorial_ bond lengths or the Rh–Rh–O_axial_ and O_axial_–C–O_axial_ bond angles between the optimized geometries of **[1(OCMe_2_)_2_]** and **[2(OCMe_2_)_2_]**.

**Figure 5:**
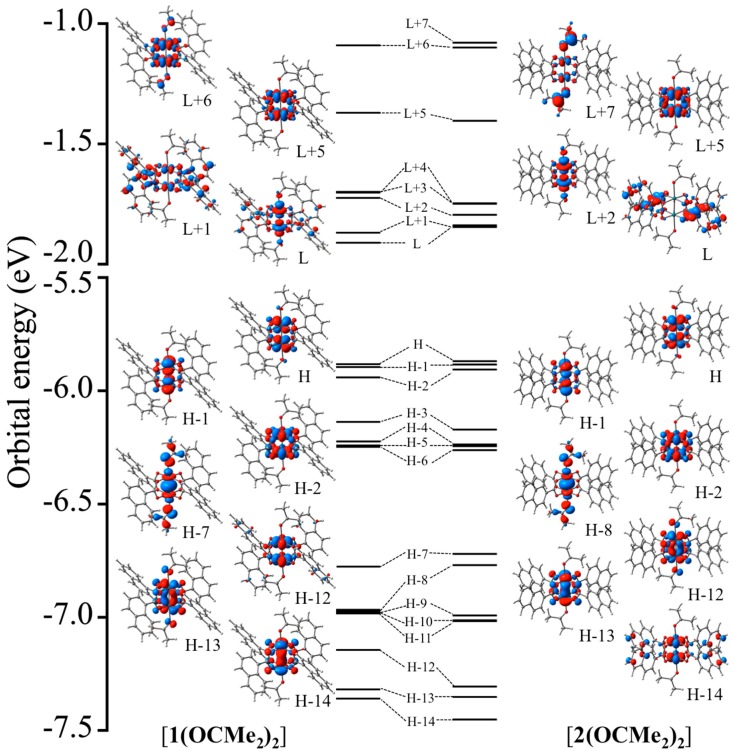
Molecular orbital (MO) energies and diagrams of **[1(OCMe_2_)_2_]** and **[2(OCMe_2_)_2_]**. Here, H and L are highest occupied MO (HOMO) and lowest unoccupied MO (LUMO), respectively.

Single-point energy calculations of the optimized geometries of **[1(OCMe_2_)_2_]** and **[2(OCMe_2_)_2_]** afforded the closed-shell singlet electronic configurations. Figure 5 shows the obtained electronic structures with selected MO diagrams. In the occupied spaces, the electronic structures between the two Rh ions, in **[1(OCMe_2_)_2_]** and **[2(OCMe_2_)_2_]**, were π^4^δ^2^σ^2^δ*^2^π*^4^ and δ^2^π^4^σ^2^δ*^2^π*^4^, respectively. Overall, the theoretical results indicated that: (i) a stable single bond between two Rh ions was formed in **[1(OCMe_2_)_2_]** and **[2(OCMe_2_)_2_]**; and (ii) slight differences of the MO ordering were present for **[1(OCMe_2_)_2_]** and **[2(OCMe_2_)_2_]**. Specifically, the orbital energies of the highest occupied MO (HOMO) and HOMO-1 of **[1(OCMe_2_)_2_]** and **[2(OCMe_2_)_2_]** were degenerate, and their MOs were assigned as two π*^2^(Rh_2_) orbitals. The δ*^2^(Rh_2_) orbitals, which had sizable orbital interactions with the *p*(O) orbitals in the *n*-NC ligands, were found in the HOMO-2. In HOMO-4 through HOMO-6, of **[1(OCMe_2_)_2_]** and **[2(OCMe_2_)_2_]**, delocalized MOs across the entire molecule or π(*naphthyl rings*) orbitals were observed. The σ^2^(Rh_2_) orbitals of **[1(OCMe_2_)_2_]**, which had sizable anti-bonding orbital interactions with the *p*(O) orbitals of the OCMe_2_ ligands, was found in HOMO-7, whereas those of **[2(OCMe_2_)_2_]** were observed in HOMO-8. In HOMO-9 through HOMO-11, of **[1(OCMe_2_)_2_]** and [2(OCMe_2_)_2_], the MOs were delocalized with *π* characters throughout the naphthyl ring moieties. The energies of two π^2^(Rh_2_) orbitals were degenerate, and their energies were almost identical for **[1(OCMe_2_)_2_]** and **[2(OCMe_2_)_2_]**. However, the orbital stabilities of the δ^2^(Rh_2_) orbitals of **[1(OCMe_2_)_2_]** and **[2(OCMe_2_)_2_]** were affected by the *n*-NC ligands; the δ^2^(Rh_2_) orbital was slightly higher in energy than the two π^2^(Rh_2_) orbitals in **[1(OCMe_2_)_2_]**, whereas the δ^2^(Rh_2_) orbital was clearly lower in energy than the two π^2^(Rh_2_) orbitals in **[2(OCMe_2_)_2_]**.

In the unoccupied MOs of **[1(OCMe_2_)_2_]** and **[2(OCMe_2_)_2_]**, the MO energies were strongly affected by the orbital interactions of the *n*-NC ligands. The lowest unoccupied MO (LUMO) and LUMO+1 of **[1(OCMe_2_)_2_]** were mainly localized on the 1-NC ligand moieties. The σ*^2^(Rh_2_) orbital, which had sizable anti-bonding orbital interactions with the *p*(O) orbitals of the OCMe_2_ ligands, was found in LUMO+2. In contrast, the σ*^2^(Rh_2_) orbital of **[2(OCMe_2_)_2_]** was found in the LUMO, where the MOs in LUMO+1 and LUMO+2 were delocalized on the Rh_2_ and 2-NC ligand moieties. The HOMO-LUMO gaps of **[1(OCMe_2_)_2_]** and **[2(OCMe_2_)_2_]** were estimated as 3.97 and 4.02 eV, respectively. Unoccupied δ*^2^(Rh_2_) and δ^2^(Rh_2_) orbitals of **[1(OCMe_2_)_2_]** were found at LUMO+5 and LUMO+6, respectively, whereas those of **[2(OCMe_2_)_2_]** were identified at LUMO+5 and LUMO+7, respectively.

### 2.4. Absorption Spectral Properties of **[1(OCMe_2_)_2_]** and **[2(OCMe_2_)_2_]**

**Figure 6:**
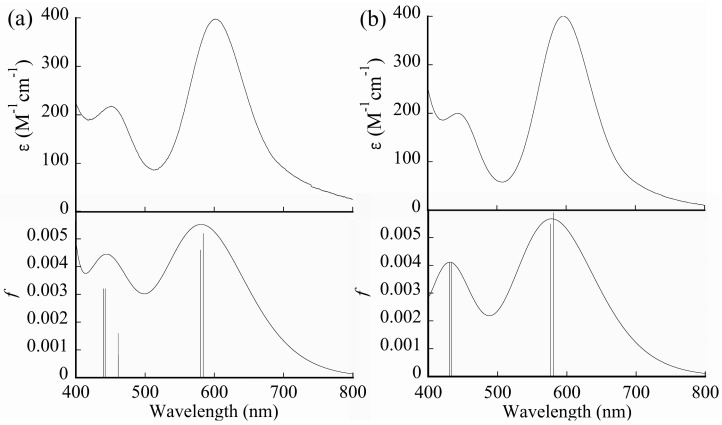
Experimental (top) and calculated (bottom) absorption spectra of: (**a**) **[1(OCMe_2_)_2_]**; and (**b**) **[2(OCMe_2_)_2_]**. Here, experimental spectra were measured in CHCl_3_:OCMe_2_ (1:1, *v*/*v*) solvent.

The absorption spectra of **[1(OCMe_2_)_2_]** and **[2(OCMe_2_)_2_]** were measured in CHCl_3_:OCMe_2_ (1:1, *v*/*v*). **[1(OCMe_2_)_2_]** and **[2(OCMe_2_)_2_]** retained their molecular structures in solution, without dissociation or exchange of axial-coordinated acetone ligands, because of the lack of coordination mobility of CHCl_3_. Figure 6 shows the absorption spectra of **[1(OCMe_2_)_2_]** and **[2(OCMe_2_)_2_]** in the visible light region. As can be seen, the obtained spectra of **[1(OCMe_2_)_2_]** and **[2(OCMe_2_)_2_]** had two absorption maxima, with similar shapes to the absorption spectra of [Rh_2_(O_2_CCH_3_)_4_(OCMe)_2_] in OCMe_2_. In particular, **[1(OCMe_2_)_2_]** showed two absorption maxima at 603 (ε = 397 M^−1^cm^−1^) and 451 (ε = 218 M^−1^cm^−1^) nm. Analogous absorption bands were also observed for **[2(OCMe_2_)_2_]**; in this case, the absorption maxima occurred at 596 (ε = 400 M^−1^cm^−1^) and 442 (ε = 200 M^−1^cm^−1^) nm. Remarkably, excitation wavelengths of **[1(OCMe_2_)_2_]** and **[2(OCMe_2_)_2_]** were almost identical to those of [Rh_2_(O_2_CCH_3_)_4_(OCMe_2_)_2_] (604 and 440 nm, respectively). Overall, any significant absorption spectral difference between **[1(OCMe_2_)_2_]** and **[2(OCMe_2_)_2_]** in the visible light region was not observed. Unfortunately, vibrational absorption bands of the *n*-NC moieties, of **[1(OCMe_2_)_2_]** and **[2(OCMe_2_)_2_]**, in the UV region could not be measured because of the overlapping absorption windows of the CHCl_3_:OCMe_2_ (1:1, *v*/*v*) solvent.

To clarify the nature of the absorption spectral features of **[1(OCMe_2_)_2_]** and **[2(OCMe_2_)_2_]** in terms of their corresponding MOs, TDDFT calculations were carried out. The calculated absorption data, such as excitation wavelengths, oscillator strengths, and assignments, of the excitation characters of **[1(OCMe_2_)_2_]** and **[2(OCMe_2_)_2_]** are summarized in Tables S1 and S2, respectively. As shown in Figure 6, the calculated absorption spectra of **[1(OCMe_2_)_2_]** and **[2(OCMe_2_)_2_]** reproduced their observed spectra quite well. Interestingly, although the shapes and intensities of the observed spectra of **[1(OCMe_2_)_2_]** and **[2(OCMe_2_)_2_]** were similar to each other, clear differences were observed for their theoretically-determined excitation characters. The low-energy absorption band of **[1(OCMe_2_)_2_]**, observed experimentally at 603 nm, was calculated to be composed of two excitations (S_0_ → S_1_ and S_2_), which were both assigned as π^2^(Rh_2_) → σ*^2^(Rh_2_) excitations. The high-energy absorption band, observed experimentally at 451 nm, was reproduced by the summation of three excitations (S_0_ → S_5_, S_6_, and S_7_). Here, the S_0_ → S_5_ excitation was assigned as δ^2^(Rh_2_) → δ*^2^(Rh_2_) and δ*^2^(Rh_2_) → δ^2^(Rh_2_) excitations, whereas the S_0_ → S_6_ and S_7_ excitations involved the π*^2^(Rh_2_) → σ*^2^(Rh_2_) excitation as the major contributor and π*^2^(Rh_2_) → δ^2^*(Rh_2_)/π*(1-NC) excitations as a minor contributor, which were considered as metal–to–metal-ligand charge transfer (MMLCT). Thus, the high-energy absorption band of **[1(OCMe_2_)_2_]** mixed the absorption characters of d–d and MMLCT transitions. Similar to **[1(OCMe_2_)_2_]**, the low-energy absorption band of **[2(OCMe_2_)_2_]**, observed experimentally at 596 nm, was composed of two excitations, which were assigned as π*^2^(Rh_2_) → σ*^2^(Rh_2_) excitations. However, the high-energy absorption band, which was experimentally observed at 442 nm, was composed of two excitations (S_0_ → S_6_, S_7_) with a π*^2^(Rh_2_) → δ*(Rh_2_) excitation character. Thus, the absorption character of **[2(OCMe_2_)_2_]** in the visible light region was comprised of only d–d transitions. These results indicate that the position of the carboxylic group in the naphthyl ring affected the absorption characters of the dirhodium complexes in the visible light region.

### 2.5. Redox Properties of **[1(OCMe_2_)_2_]** and **[2(OCMe_2_)_2_]**

**Figure 7:**
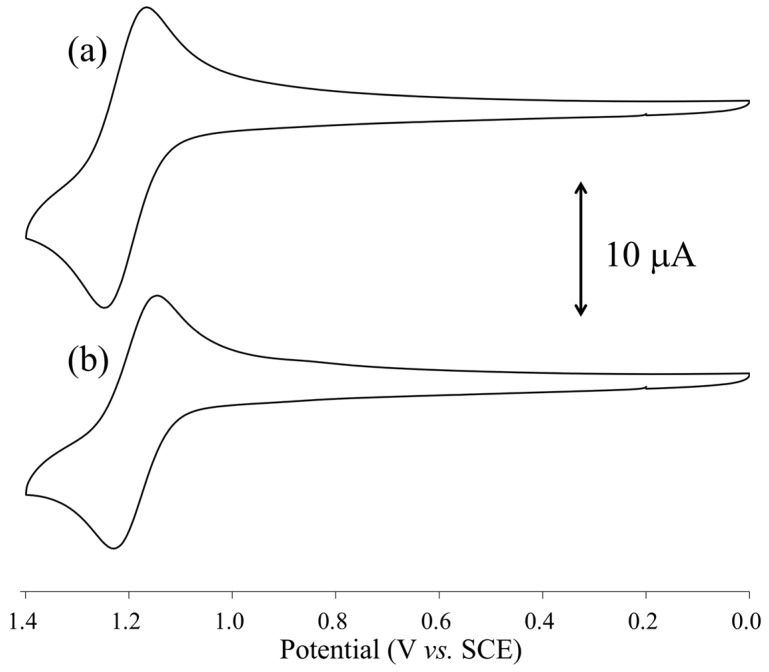
Cyclic voltammetry (CV) diagrams, in CHCl_3_:OCMe_2_ (1:1, *v*/*v*) solvent, of: (**a**) **[1(OCMe_2_)_2_]**; and (**b**) **[2(OCMe_2_)_2_]**.

The electrochemical properties of **[1(OCMe_2_)_2_]** and **[2(OCMe_2_)_2_]** in dried CHCl_3_:OCMe_2_ (1:1, *v*/*v*) were investigated via cyclic voltammetry (CV), and the resultant CV curves are shown in Figure 7. In the positive region, **[1(OCMe_2_)_2_]** and **[2(OCMe_2_)_2_]** had one reversible wave at 1.21 and 1.19 V vs. SCE, respectively, which were assigned as the 1*e*^–^ oxidation of the Rh_2_ center in **[1(OCMe_2_)_2_]** and **[2(OCMe_2_)_2_]**. Compared with the redox potential of [Rh_2_(O_2_CPh)_4_(OCMe_2_)_2_] (1.17 V vs SCE) and [Rh_2_(O_2_CCH_3_)_4_(OCMe_2_)_2_] (1.14 V vs SCE) in CHCl_3_:OCMe_2_, those of **[1(OCMe_2_)_2_]** and **[2(OCMe_2_)_2_]** were slightly shifted in the positive direction. These results indicate that the electron-withdrawing abilities of the *n*-NC ligands for the dinuclear core were slightly stronger than those of the O_2_CPh and OCMe ligands. The uDFT calculations of **[1(OCMe_2_)_2_]^+^** and **[2(OCMe_2_)_2_]^+^** indicated that one electron was removed from the π* orbital of each Rh_2_ center.

## 3. Experimental

### 3.1. Materials and Instruments

[Rh_2_(O_2_CCH_3_)_4_(H_2_O)_2_] was prepared according to the literature procedures [27,28]. All the reagents were purchased from commercial sources (Wako Co. Ltd., Osaka, Japan, and TCI, Tokyo, Japan) and used without further purification. ^1^H NMR spectra were measured with a JEOL-ECS 500SS spectrometer (Tokyo, Japan ) in acetone-*d*_6_ (tetramethylsilane was used as internal reference standard). The infrared spectra were measured using a JASCO FT-IR 660-plus spectrometer (Tokyo, Japan) in KBr disks at room temperature. Raman spectra were recorded on a Renishaw Raman system 2000 spectrometer (Gloucestershire, UK) equipped with a He-Ne laser (633 nm) as the excitation source. Electrospray ionization mass spectrometry (ESI-MS) was conducted using a Bruker micrOTOF spectrometer (Billerica, MS, USA). Here, sodium formate was used as calibration standard. Elemental analyses for carbon and hydrogen were carried out using a Yanaco CHN CORDER MT-6 installed at Shimane University, Japan. UV-visible absorption spectrum was measured in CHCl_3_/acetone (1:1) using a JASCO V-670 spectrometer (Tokyo, Japan). X-ray powder diffraction (XRPD) analyses were performed with a RIGAKU MiniFlex II diffractometer (Tokyo, Japan). Thermogravimetric analyses were conducted with a MAC Science TG-DTA 2000S (Billerica, MS, USA), with a heating rate of 4 K/min under air atmosphere. Cyclic voltammetry (CV) was measured in dried CHCl_3_/acetone (1:1), containing tetra-*n*-butylammonium hexafluorophosphate (TBAPF_6_) as an electrolyte, using BAS ALS-DY 2325 electrochemical analyzer (Tokyo, Japan). A glassy carbon disk (1.5 mm radius), Pt wire, and saturated calomel electrode (SCE) were used as the working, counter, and reference electrodes, respectively.

### 3.2. Materials and Instruments Synthesis of [Rh_2_(1-NC)_4_(OCMe_2_)_2_] (**[1(OCMe_2_)_2_]**)

A mixture of [Rh_2_(O_2_CCH_3_)_4_(H_2_O)_2_] (47.8 mg, 0.10 mmol) and 1-HNC (687.3 mg, 4.00 mmol) in degassed EtOH (10.0 mL) was sealed in an autoclave under Ar, and then heated at 413 K for 7 h. The resultant precipitate was filtered with a membrane filter, washed with EtOH, and dissolved in acetone. The resultant green solution was evaporated to almost dryness, and the green precipitate formed was collected on membrane filter and dried under vacuum. Yield: 91.2 mg (90.6%). Anal. Calc. for C_50_H_40_O_10_Rh_2_: C 59.66%, H 4.01%. Found: C, 59.57%; H, 4.15%. ^1^H NMR (500 MHz, acetone-*d*_6_, 298K): δ = 8.63 (d [7.5 Hz], 4H), 7.98 (d [8.0 Hz], 4H), 7.89 (m, 8H), 7.45 (m, 12H). ESI-MS: Calcd. for [M + Na]^+^ 912.9786 *m*/*z*; Found 912.9783. IR (KBr disk, cm^−1^): 3048 (vw), 2923 (vw), 1680 (s), 1599 (w), 1561 (s), 1509 (w), 1461 (w), 1406 (s), 1375 (vs), 1355 (s), 1256 (w), 1234 (w), 1153 (w), 872 (w), 818 (w), 784 (s), 664 (m), 552 (m), 516 (m), 488 (m).

### 3.3. Synthesis of [Rh_2_(2-NC)_4_(OCMe_2_)_2_] (**[2(OCMe_2_)_2_]**)

A similar synthetic procedure to that of [1(OCMe_2_)_2_] was used for the synthesis of [2(OCMe_2_)_2_], but 2-HNC was used instead of 1-HNC. Yield: 89.7 mg (89.1%). Anal. Calc. for C_50_H_40_O_10_Rh_2_: C 59.66, H 4.01%. Found: C 59.35%, H 3.82%. ^1^H NMR (500 MHz, acetone-*d*_6_, 298K): δ = 8.47 (s, 4H), 7.96 (td [9.75 Hz], 8H), 7.82 (d [8.0 Hz], 4H), 7.78 (d [9.0 Hz], 4H), 7.49 (m, 8H). ESI-MS: Calcd. for [M + Na]^+^ 912.9786 *m*/*z*; Found 912.9775. IR (KBr disk, cm^−1^): 3052 (w), 1680 (m), 1602 (m), 1563 (m), 1469 (m), 1400 (vs), 1238 (w), 797 (m), 783 (m), 765 (m), 648 (w), 608 (w), 475 (w).

### 3.4. Single Crystal X-Ray Diffraction Analyses

Single crystal X-ray diffraction data of **[1(OCMe_2_)_2_]** and **[2(OCMe_2_)_2_]** were collected at 150 K on a RIGAKU Saturn 724 CCD system equipped with a Mo rotating-anode X-ray generator with Monochromated Mo-K radiation (λ = 0.71075 Å), installed in Kanagawa University, and were processed using the CrystalClear program (RIGAKU). The structures of **[1(OCMe_2_)_2_]** and **[2(OCMe_2_)_2_]** were solved by direct methods SIR-2004 [29] and IL MILIONE [30], respectively, and refined using the full-matrix least-squares technique F^2^ with SHELXL2014 [31], equipped in the CrystalStructure 4.2.1 software (RIGAKU) (Tokyo, Japan). Non-hydrogen atoms were refined with anisotropic displacement, and almost all of hydrogen atoms were located at the calculated positions and refined as riding models. Crystal data, as well as the details of data collection and refinement for **[1(OCMe_2_)_2_]** and **[2(OCMe_2_)_2_]**, are summarized in Table 1 and can be obtained as CIF files from Cambridge Crystallographic Data Center (CCDC). Deposition numbers of **[1(OCMe_2_)_2_]** and **[2(OCMe_2_)_2_]** are CCDC-1875465 and CCDC-1875466, respectively.

### 3.5. Details of Theoretical Calculations

All the density functional theory (DFT) calculations were performed using the Gaussian 09 C.01 program package [26]. The hybrid DFT functional method, B3LYP, with the Los Alamos effective core potential (ECP) basis set, LANL08(f), for Rh atoms; the Dunning’s augmented correlation consistent basis set, aug-cc-pVDZ, for O atoms; and correlation consistent basis set, cc-pVDZ, for the other atoms, were employed. Initial molecular geometries of **[1(OCMe_2_)_2_]** and **[2(OCMe_2_)_2_]** were obtained from CIF files. All molecular geometries were fully optimized in solvent media. The solvent effect of acetone was considered by the integral equation formalism polarizable continuum model (IEF-PCM). Here, the cavity surface areas of **[1(OCMe_2_)_2_]** and **[2(OCMe_2_)_2_]** were set at 966.91 and 977.31 Å^2^, respectively. The spin-allowed adiabatic excitations were calculated by the time-dependent DFT (TDDFT) method. The simulated absorption spectra were drawn by using the GaussView 5.0 program (Wallingford, CT, USA). with a half-width at half-height value of 0.250 eV (2016.4 cm^−1^). In terms of the calculation of oxidized species of **[1(OCMe_2_)_2_]** and **[2(OCMe_2_)_2_]**, we used the unrestricted broken-symmetry approach for determining the open-shell electronic structures of **[1(OCMe_2_)_2_]^+^** and **[2(OCMe_2_)_2_]^+^**.

## 4. Conclusions

In this article, two *n*-naphthate-bridged dirhodium complexes, **[1(OCMe_2_)_2_]** and **[2(OCMe_2_)_2_]**, were solvothermally synthesized and characterized via single crystal and powder X-ray diffraction analyses, infrared spectroscopy, ESI-MS, ^1^H NMR spectroscopy, and elemental analyses. Single crystal structure analyses proved that four naphthalene rings, in **[1(OCMe_2_)_2_]**, individually formed intermolecular π–π stacking interactions with the naphthalene rings of four neighboring **[1(OCMe_2_)_2_]** moieties; whereas those in **[2(OCMe_2_)_2_]** only formed intermolecular hydrogen bonding interactions in its packing structure. From this result it is expected that **[1(OCMe_2_)_2_]** and **[2(OCMe_2_)_2_]** could be used as the robust building blocks for coordination polymers and MOFs with supramolecular interactions. Electronic structure analyses, by means of DFT calculations, indicated that: (i) both complexes had a single bond between two Rh ions, and (ii) slight electronic structure differences between two Rh ions occurred in **[1(OCMe_2_)_2_]** (π^4^δ^2^σ^2^δ*^2^π*^4^) and **[2(OCMe_2_)_2_]** (δ^2^π^4^σ^2^δ*^2^π*^4^). Although the observed shapes and intensities of the absorption spectra were nearly the same between **[1(OCMe_2_)_2_]** and **[2(OCMe_2_)_2_]**, their calculated excitation characters differred slightly. **[1(OCMe_2_)_2_]** showed d–d and MMLCT excitations in the visible light region, whereas **[2(OCMe_2_)_2_]** showed only d–d excitations in the same region. That is, the position of the carboxylic group, attached to the naphthalene ring, had hardly any effect on the electronic structures and absorption properties of *n*-naphthoate-bridged dirhodium complexes. The one-electron oxidation potentials of **[1(OCMe_2_)_2_]** and **[2(OCMe_2_)_2_]** were slightly shifted to the positive-side, compared with those of [Rh_2_(O_2_CPh)_4_(OCMe_2_)_2_] and [Rh_2_(O_2_CCH_3_)_4_(OCMe_2_)_2_], because of the electron-withdrawing effects of naphthalene rings. This knowledge is also important for the development of dirhodium complexes that have the ability for oxidation catalysis. We believe that the results of this study become the foundation for the further development of acene carboxylate-coordinated paddlewheel-type dirhodium complexes.

## Figures and Tables

**Table 1:** Crystallographic data for complexes **[1(O_2_CMe_2_)_2_]** and **[2(O_2_CMe_2_)_2_]**.

Complex	[1(OCMe_2_)_2_]	[2(OCMe_2_)_2_]
Crystal size (mm^3^)	0.18 × 0.14 × 0.14	0.14 × 0.04 × 0.04
Chemical formula	C_50_H_40_O_10_Rh_2_	C_50_H_40_O_10_Rh_2_
*T* (K)	150	150
Formula weight (g/mol)	1006.64	1006.64
Crystal system	Triclinic	Monoclinic
Space group	*P*-1	*P* 2_1_/*c*
*a* (Å)	10.480(5)	10.195(7)
*b* (Å)	10.960(5)	17.537(10)
*c* (Å)	11.441(6)	13.264(9)
(°)	98.215(4)	90
(°)	115.975(3)	110.979(10)
(°)	110.223(2)	90
*V* (Å^3^)	1038.9(9)	2214(2)
*Z*	1	2
*D*_calc_(g/cm^3^)	1.609	1.510
*F*(000)	510	1020
Final *R*_1_ indices [*I* > 2σ(*I*)]	*R*_1_ = 0.0345, *wR*_2_ = 0.0795	*R*_1_ = 0.0947, *wR*_2_ = 0.2225
*R* indices (all data)	*R*_1_ = 0.0368, *wR*_2_ = 0.0810	*R*_1_ = 0.1195, *wR*_2_ = 0.2445
Goodness of fit (GOF) on *F*^2^	1.094	1.215

**Table 2:** Selected (averaged) structural parameters of crystal structures of **[1(O_2_CMe_2_)_2_]**, **[2(O_2_CMe_2_)_2_]**, and [Rh_2_(O_2_CCF_3_)_4_(O_2_CMe)_2_].

	[1(OCMe_2_)_2_]	[2(OCMe_2_)_2_]	[Rh_2_(O_2_CCF_3_)_4_(O_2_CMe)_2_]
Rh–Rh	2.374	2.395	2.406
Rh–O_equatorial_	2.035	2.040	2.036
Rh–O_axial_	2.296	2.303	2.252
Rh–Rh–O_equatorial_	88.18	88.19	87.97
Rh–Rh–O_axial_	175.51	178.54	175.67
	125.52	125.03	128.93
References	This study	This study	[25]

## Supplementary Materials

The following are available online, Figure S1: ESI-MS of [1(OCMe_2_)_2_].; Figure S2: ESI-MS of [2(OCMe_2_)_2_].; Figure S3: TG curve of [1(OCMe_2_)_2_].; Figure S4: TG curve of [2(OCMe_2_)_2_].; Figure S5: Optimized geometries of (a) [1(OCMe_2_)_2_] and (b) [2(OCMe_2_)_2_]; Table S1: Calculated excitation wavelengths, oscillator strengths, and excitation characters for [1(OCMe_2_)_2_]. (Here, H and L are HOMO and LUMO, respectively).; and Table S2: Calculated excitation wavelengths, oscillator strengths, and excitation characters for [2(OCMe_2_)_2_]. (Here, H and L are HOMO and LUMO, respectively).
