# Supplementary material for: Synthesis, Characterization, Absorption Properties, and Electronic Structures of Paddlewheel-Type Dirhodium(II) Tetra-μ-(n-naphthoate) Complexes: An Experimental and Theoretical Study
Source: Molecules. 2019 Jan 27;24(3):447. doi: 10.3390/molecules24030447 (PMC6384905; doi:10.3390/molecules24030447)
Supplement: Supplementary file 1 [file molecules-24-00447-s001.pdf]

Supporting Information

Synthesis, Characterization, Absorption Properties, and Electronic Structures of Paddlewheel-Type Dirhodium(II) Tetra-μ-(*n*-naphthoate) Complexes: An Experimental and Theoretical Study

Yusuke Kataoka,[a],* Raiki Fukumoto,[a] Natsumi Yano,[a] Daiki Atarashi,[a] Hidekazu Tanaka, [a] Tatsuya Kawamoto, [b] Makoto Handa[a],*

[a] Department of Chemistry, Interdisciplinary Graduate School of Science and Engineering, Shimane University, 1060 Nishikawatsu, Matsue, Shimane 690-8504, Japan.

[b] Department of Chemistry, Faculty of Science, Kanagawa University, 2946 Tsuchiya, Hiratsuka, Kanagawa 259-1293, Japan.

Corresponding address: kataoka@riko.shimane-u.ac.jp (Y. Kataoka)

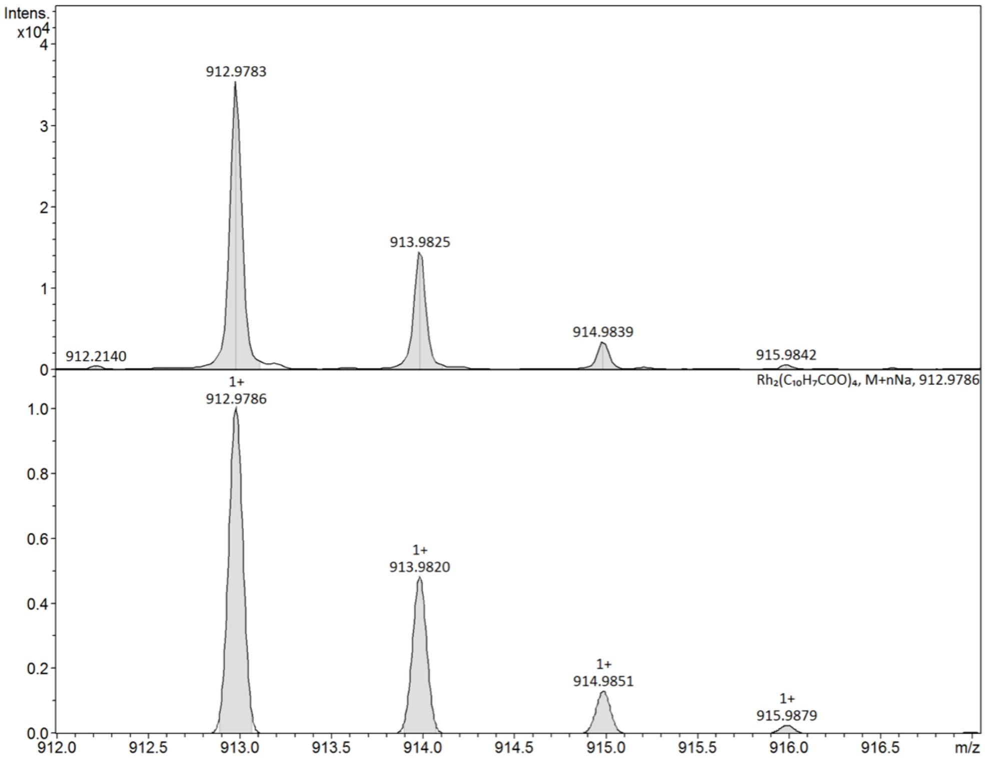

Figure S1. ESI-MS of **[1(OCMe$_2$)$_2$]**.

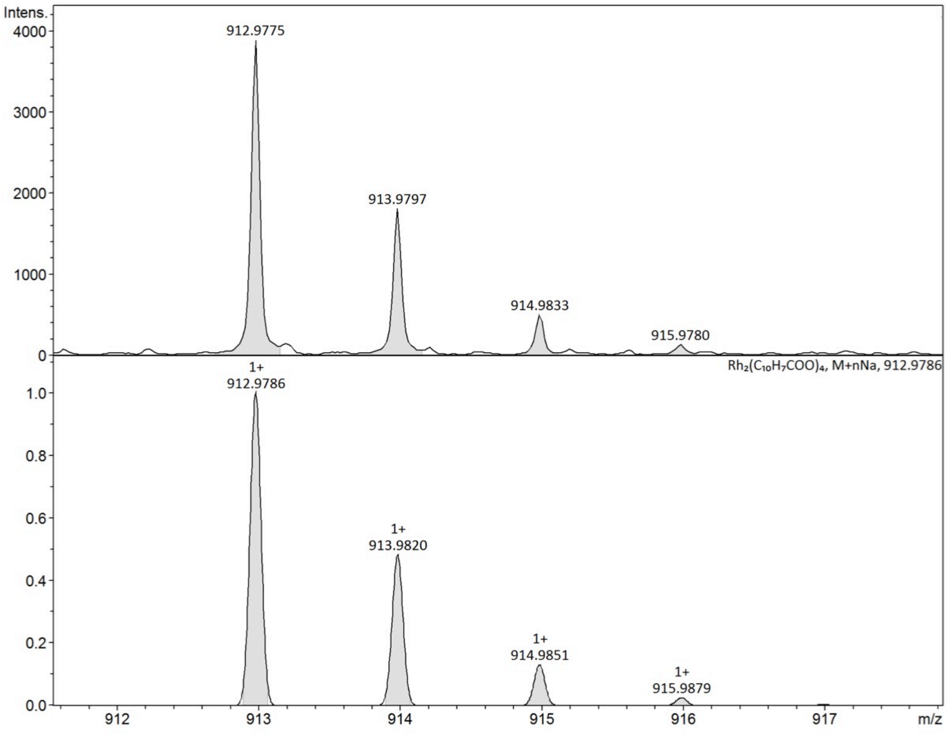

Figure S2. ESI-MS of **[2(OCMe$_2$)$_2$]**.

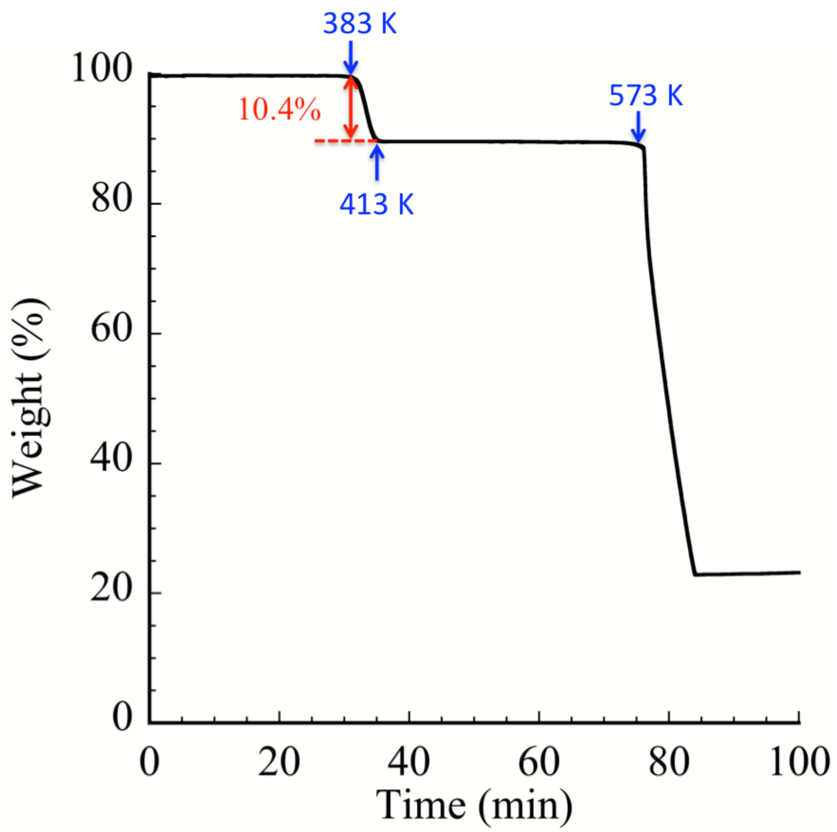

Figure S3. TG curve of **[1(OCMe$_2$)$_2$]**.

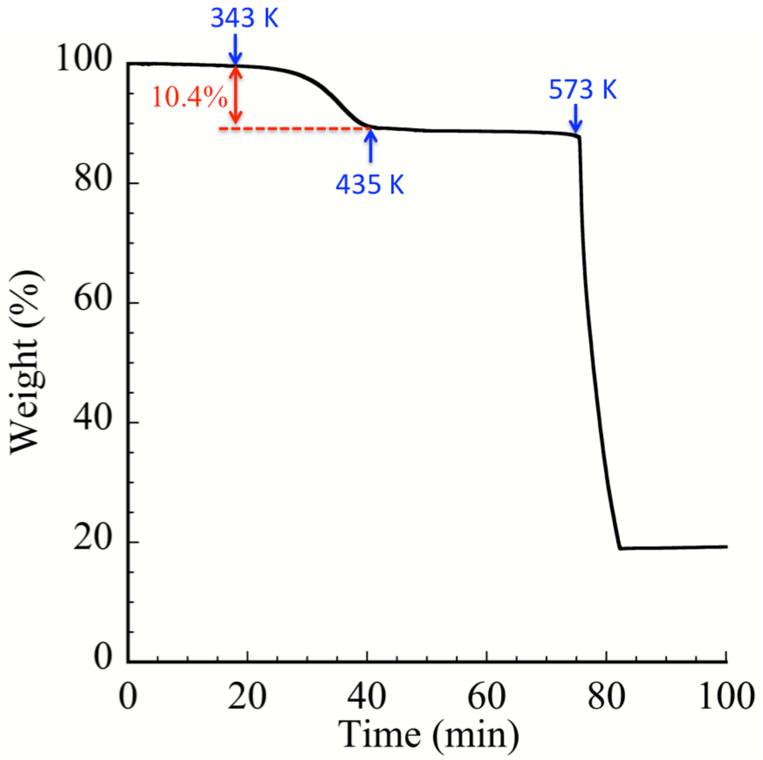

Figure S4. TG curve of **[2(OCMe$_2$)$_2$]**.

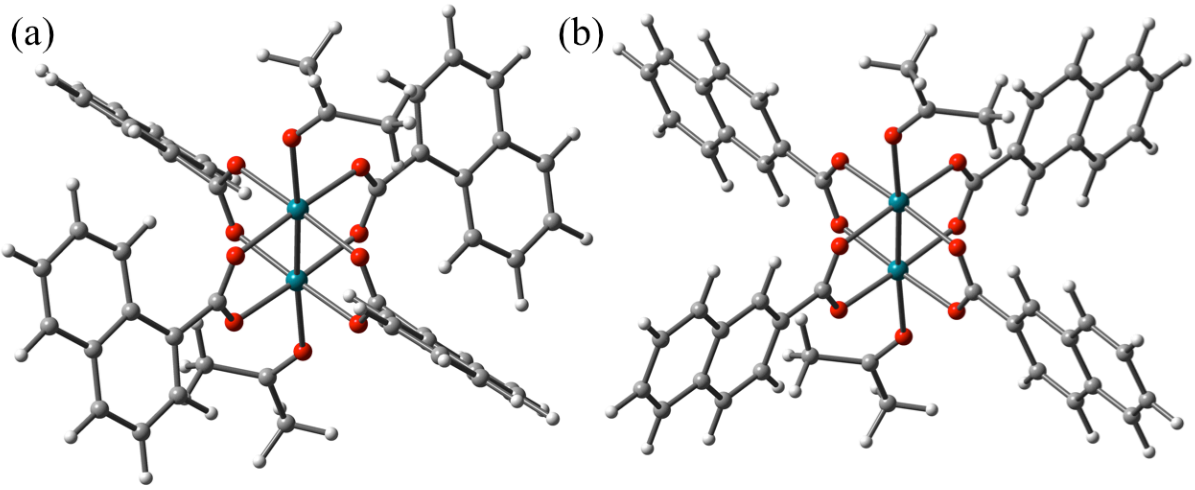

Figure S5. Optimized geometries of (a) **[1(OCMe$_2$)$_2$]** and (b) **[2(OCMe$_2$)$_2$]**.

Table S1. Calculated excitation wavelengths, oscillator strengths, and excitation characters for **[1(OCMe$_2$)$_2$]**. (Here, H and L are HOMO and LUMO, respectively).

State	Wavelength (nm)	Oscillator strengths (*f*)	Major contribution
S_1	584.1	0.0052	H → L (53%), H → L+4 (30%)
S_2	580.3	0.0046	H-1 → L (52%), H-1 → L+4 (29%)
S_5	461.1	0.0016	H-2 → L+6 (56%), H-12 → L+5 (16%)
S_6	442.4	0.0032	H → L+5 (51%), H → L+1 (34%)
S_7	439.9	0.0032	H-1 → L+5 (51%), H-1 → L+1 (32%)

Table S2. Calculated excitation wavelengths, oscillator strengths, and excitation characters for **[2(OCMe$_2$)$_2$]**. (Here, H and L are HOMO and LUMO, respectively).

State	Wavelength (nm)	Oscillator strengths (*f*)	Major contribution
S_1	580.7	0.0059	H → L+2 (84%)
S_2	576.5	0.0055	H-1 → L+2 (83%)
S_6	433.1	0.0041	H → L+5 (84%)
S_7	430.2	0.0041	H-1 → L+5 (85%)
